# Public health quality indicators as a prioritization and leadership tool: a scoping review of their role in health system transformation

**DOI:** 10.1093/eurpub/ckaf174

**Published:** 2026-01-23

**Authors:** Christos Triantafyllou, Anastasia Ntikoudi, Anastasia Papachristou, Vion Psiakis, Valter R Fonseca, Joao Breda

**Affiliations:** WHO Office on Quality of Care and Patient Safety, Athens, Greece; WHO Office on Quality of Care and Patient Safety, Athens, Greece; WHO Office on Quality of Care and Patient Safety, Athens, Greece; WHO Office on Quality of Care and Patient Safety, Athens, Greece; WHO Office on Quality of Care and Patient Safety, Athens, Greece; WHO Office on Quality of Care and Patient Safety, Athens, Greece

## Abstract

Public health indicators serve as vital monitoring tools of population’s health while assisting policy makers in their leadership role while guiding policy decisions. Standardized indicator development continues to face substantial obstacles regarding their conceptual definition, methodological precision, and national compatibility. The aim of this review was to combine academic and institutional literature to assess the application of quality indicators in public health settings. A scoping review of the existing literature on public health quality indicators was conducted. The search was performed in PubMed, EMBASE, and CINAHL databases. Eleven publications were included, and the extracted data were organized in a structured table. Research findings showed that indicators must retain a balance between usefulness, national context adaptability and standardized frameworks. The ECHI, EUHPID, and PAHO’s frameworks established systematic methods to organize indicators and create measurement systems. Subnational programs highlighted that data quality and coverage remained insufficient. Public health indicators serve as essential tools for tracking population health status while assisting in policy decisions. The practical application of indicators depends on their methodological soundness, ethical approach and their practical implementation possibilities. Research demonstrates that public health indicators require continuous investment regarding their technical infrastructure and conceptual frameworks. Future research should include indicator policy impact assessment, framework improvement and real-time public health system assessment.

## Introduction

Public health is a broad and developing field focused on diseases prevention, extending life expectancy and enhancing population health. The definitions established by Charles-Edward A. Winslow and Sir Donald Acheson continue to influence contemporary understanding of the field [1, 2]. The definition of Winslow still remains popular as it focuses on collective societal initiatives, yet Acheson’s 1988 definition added emphasis on equity and policy integration [3]. The 2011 concept paper from the World Health Organization (WHO) Regional Office for Europe utilized Acheson’s definition as a practical reference after evaluating perspectives from important public health experts [4].

The Pan American Health Organization (PAHO) asserts that the enhancement of public health is achieved by implementing its established principles. To fully understand and identify the different components of public health, such as activities, intelligence, systems, skills, and competencies, it is crucial to have a clear and precise definition [5]. A well-defined definition of public health allows individuals engaged in, benefiting from, or studying the field to effectively classify its elements, comprehend its complexities, and work towards its improvement.

Public health has encountered multiple challenges during the last century which include both the COVID-19 pandemic [6] and the increasing prevalence of non-communicable diseases (NCDs) [7]. NCDs have been the main public health concern worldwide for multiple decades causing morbidity, mortality and inequities to rise. These distinct health threats have now merged into a ‘syndemic,’ a new term that describes the mechanism that NCDs and infectious diseases work together to deteriorate health outcomes, specifically in low- and middle- income countries [8].

Quality indicators serve as a standard tool for the evaluation of healthcare services, particularly in primary health care as emphasized recently by WHO [9]. These quality indicators have been linked to performance and care provision; however, a focus on core public health functions such as health promotion and population health management is still not fully available. Public health indicators are quantifiable measures that reflect the health status of a population and determinants influencing it [10]. The lack of standardization in quality terminology between public health and healthcare settings creates conceptual ambiguity throughout the literature [11, 12]. The Public Health Quality Forum (2008) established that public health quality represents the extent to which health policies, programs, services, and research focused on population achieve the desired health outcomes for the population addressed [13]. The Donabedian framework guides public health quality measurement through three major categories which include structure, process, and outcome measures. This threefold framework establishes a clear distinction between general public health indicators and public health quality indicators adopted in this scoping review [14].

The current scoping review aims to combine existing academic and institutional knowledge regarding the development of public health indicators as well as their classification systems and frameworks. The review examines public health indicator design and implementation through standardized indicator frameworks that are effective and compatible.

## Methods

A literature review in a scoping review design was conducted to examine the existing literature regarding public health quality indicators [15, 16].

This scoping review was performed in accordance with the PRISMA-ScR guidelines in order to promote transparency, methodological rigor, and reproducibility. The PRISMA-ScR checklist served as a fundamental tool throughout the review, guiding the development of research questions as well as the data extraction process and synthesis. By following these protocols, a structured approach was ensured for this review, outlining the research strategy, inclusion criteria, and data extraction techniques. The review protocol was not submitted in any publicly accessible registry. The selection of studies consisted of two stages which started with title and abstract screening and was followed by full-text eligibility assessment. Two reviewers independently conducted the record screening process and resolved disagreements through discussion or by consulting with a third reviewer. Data were extracted with the use of a standardized table in order to collect crucial information from every source including publication date, geographical/institutional information, quality indicator purposes, methodological details, and important findings. The literature underwent thematic analysis which revealed essential domains among the selected sources.

**Figure 1.**
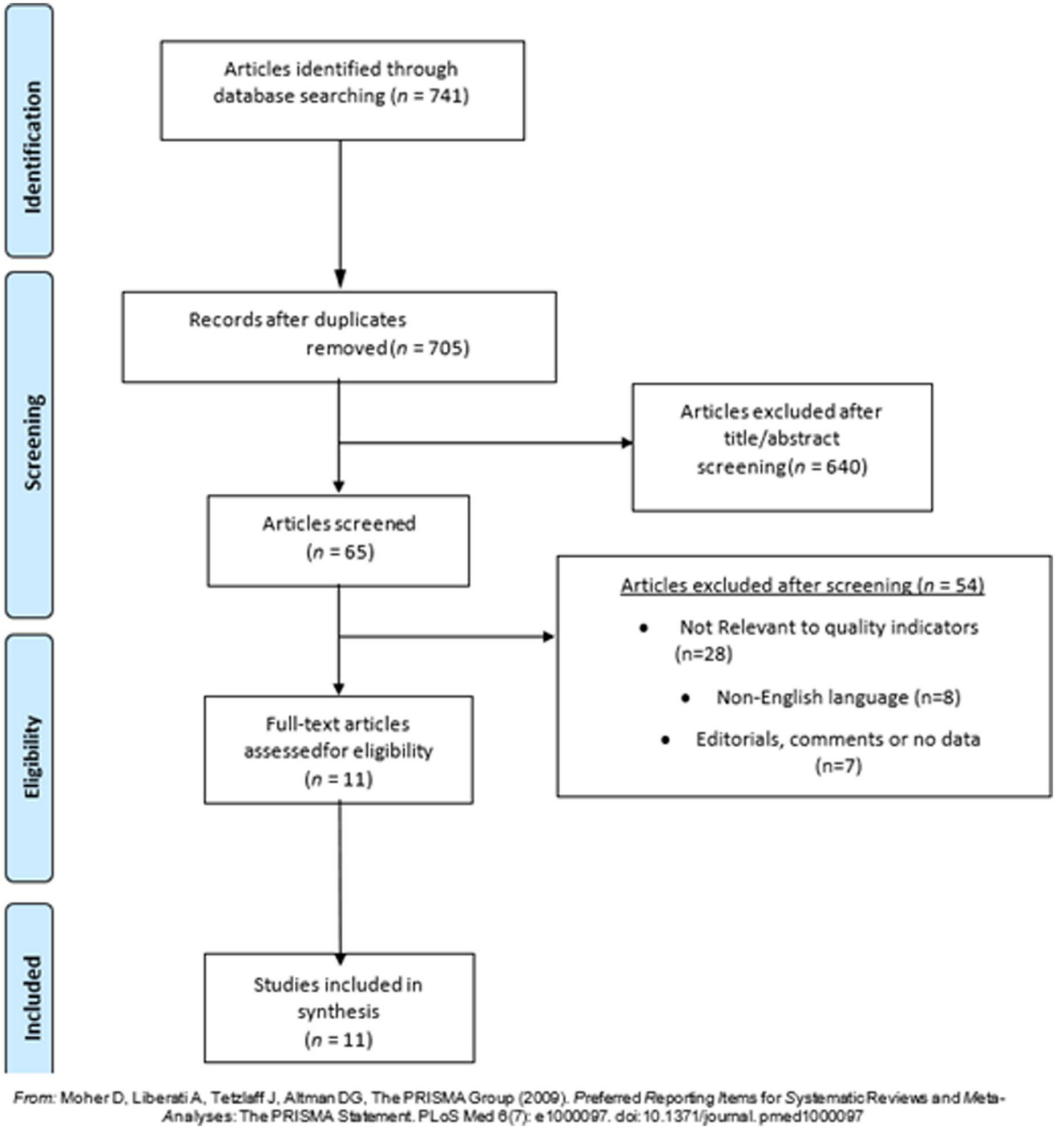
Flow of information through the steps of the scoping review process, including identification, screening, eligibility, and inclusion.

A comprehensive search was carried out from 2000 until 2025 across electronic databases, including PubMed, EMBASE and Cinahl. The search strategy used MeSH terms such as (‘public health’ OR ‘health services’ OR ‘community health’) AND (‘quality indicators’ OR ‘performance indicators’ OR ‘quality measures’) AND (‘evaluation’ OR ‘assessment’ OR ‘measurement’). A combination of Boolean operators with manual bibliography screening and institutional website searches from the WHO European Commission and Pan American Health Organization (PAHO) helped identify additional references. The database searches produced 741 relevant articles for review. The abstract screening process resulted in 65 articles that underwent full-text review. The final stage of analysis resulted in choosing 11 essential publications that underwent thorough thematic evaluation based on the established inclusion-exclusion criteria. The final selection of publications included technical reports from international public health organizations and peer-reviewed scientific articles (Fig. 1).

The research included evidence-based studies, conceptual analyses, and institutional reports about public health indicators’ design, implementation, classification, and evaluation process. The research examined quality indicator frameworks and methodological validity along with regional implementation difficulties, ethical issues, and policy relevance. The review excluded research focusing only on clinical or individual indicators that did not focus on public health, opinion pieces, and editorials.

## Results

This scoping review processed 11 peer-reviewed papers and official reports to investigate quality indicator development, implementation, and evaluation processes in public health (Table 1). The following section presents study-specific findings which fit into seven thematic domains. Each section highlights a specific area of development or challenge in the application of public health indicators.

**Table 1.** Structured table of reviewed studies.

Study	Year	Context	Type and purpose	Methodological features	Key findings
Verschuuren *et al.* [17]	2013	EU/ECHI project	Comprehensive EU-wide indicator set (ECHI)	Pilot implementation, classification into three readiness levels, guidelines for implementation at national level were developed	88 Indicators defined; partial national uptake; methodological inconsistencies and data availability issues; need for sustained EU-level governance and funding
Kramers [18]	2005	EU/ECHI-2	Conceptual and practical development of indicator list established by the ECHI-1 project	Longlist/shortlist process; stakeholder input; rigid protocol for the establishment of the shortlist	82 Shortlisted indicators; concept of ‘user-windows’ that allow for the interest-oriented selection of subsets of indicators; web-based application for the interconnection of ECHI, Eurostat, WHO-Europe and the OECD indicators
Etches *et al.* [10]	2006	Canada/CIHR-IPPH conceptual framework	Population health conceptual framework	Determinants-based model; ethical analysis; literature synthesis	Indicators should perform functions such as advocacy, policy guidance, public accountability responsibilities; this framework acts as a basis for organizing indicators of public health
Flowers *et al.* [19]	2005	UK NHS	Performance indicator design and presentation	Critique of league tables; introduced control charts and funnel plots	Established criteria combined with the correct indicator presentation methods can assist in the creation of more appropriate indicators
PAHO and WHO [20]	2018	Latin America/Global	Indicator framework guide for public health	Categorization by purpose; core attributes listed	Conceptual and practical considerations for selecting and calculating health indicators were examined
Wilkinson *et al.* [21]	2007	EU/ISARE II	Regional indicator feasibility study	Pilot across 13 countries using 38 indicators	Mortality data were consistent; behavior indicators were limited; subnational disparities were identified
Surján *et al.* [22]	2006	EU/WHO, OECD, ECHI databases	Semantic modeling of health indicators	Semantic web technology to create a formal system which united 19 indicators between EU/WHO, OECD, ECHI databases	Enhanced system interoperability; challenges with dynamic relationships and relational complexity
Gaeta *et al.* [23]	2017	EU countries	System performance evaluation via indicators	Analysis of Beveridge, Bismarck and Mixed systems through 10 performance indicators	Health expenditure linked to outcomes more than system type; economic investments proved to be the primary performance-determining factor
Chinchilla-Rodríguez *et al.* [24]	2014	Latin America	Scientific output vs. public health outcomes	Analysis of bibliometric, socioeconomic and health indicators of the 10 Latin American producers of documents	English-language bias affects citations; no regular relationship between research production and population wellness
Vardoulakis *et al.* [25]	2016	EU/EURO-HEALTHY	Environmental and social health indicators for PHI	Delphi process; pilot study	Indicators reflect urban inequities; environmental factors linked to chronic disease death rates
Bauer *et al.* [26]	2006	EU/EUHPID	Theoretical health development model for indicator classification	Salutogenic-pathogenic framework; applied to ECHI shortlist; structured across health status, individual & environmental determinants	Coherent framework for interpretation, classification and monitoring of a wide range of public health and health promotion interventions

### Strategic frameworks for public health quality indicator development

Two research studies focused on the development of standardized indicators for population monitoring purposes. Verschuuren *et al.* [17] and Kramers [18] identified core components through the European Community Health Indicators (ECHI) initiative. Verschuuren *et al.* [17] compiled an indicator shortlist with 88 entries including 67 ready for use and 14 in development and 13 under construction. The pilot study, involving 25 nations, demonstrated methodological inconsistencies and data availability problems which supported the need for sustained EU-level governance and funding.

The ECHI Phase II project team led by Kramers [18] used a health determinants-based framework to reduce 400 potential indicators down to 82 strong candidates. The authors created four essential categories including demographics/socioeconomic situation, health status, determinants of health and health systems. During this project, a web-based application was established in which the ECHI indicators were listed, with their definitions, along with the indicators used by Eurostat, WHO Europe, and the OECD. The authors concluded that despite the importance of the development and improvement of indicator definition, each Member State should develop an appropriate and sustainable data collection system.

### Theoretical and ethical approaches to indicator selection and use

Etches *et al.* [10] proposed a framework that highlighted indicators not only as measurement tools but also for advocacy, public accountability, and policy support. The framework proposed focuses on the determinants of individual and population health and acts as a basis for organizing indicators of population health.

The Pan American Health Organization and WHO [20] provided a framework for developing and implementing public health indicators. The authors organized indicators based on their functions including descriptive, predictive, and evaluative categories while describing that indicators should possess feasibility, validity, clarity, and replicability. This report examined the conceptual and practical considerations for selecting and calculating health indicators. Furthermore, it highlighted how public health indicator’s function can convert data into useful information, a function that enables policy guidance and health system decision-making.

The EUHPID Health Development Model [26] organized public health indicators through health development principles. This framework included physical health indicators, mental and social well-being indicators, and focused on health promotion principles and health perspectives. These three elements of the health development model translated into three main categories of indicators such as health, individual, and environmental determinants of health indicators. This model delivered a coherent framework for interpretation, classification and monitoring of a wide range of public health and health promotion interventions. The authors concluded that the model and its application highlight the need for systematic salutogenic indicator development in the field of public health.

### Indicator presentation and methodological standards

Flowers *et al.* [19] demonstrated that league tables combined with rankings present misleading performance results because of statistical flaws and unpredictable data patterns. The researchers introduced 20 criteria to evaluate indicators’ robustness and recommended control charts and funnel plots because these visualization methods are characterized by valid construction and intuitive display. The research demonstrated that the established criteria combined with the correct indicator presentation methods can assist in the creation of more appropriate indicators.

### Regional and subnational monitoring initiatives

The importance of public health indicators at both regional and urban levels became evident through the work of Wilkinson *et al.* [21] and Vardoulakis *et al.* [25]. The ISARE II project analysed 38 indicators across 13 EU countries [21]. The authors stated that mortality information was widely available, however, data on important health topics such as obesity encountered problems with coverage and standardization. The research highlighted the need to establish a European-wide system in order to collect data on major public health issues at subnational level such as smoking and obesity [21].

Vardoulakis *et al.* [25] used a Delphi-derived set of 42 indicators to build a Population Health Index (PHI) through their EURO-HEALTHY project research which comprised 10 European metropolitan regions. The researchers examined Greater London Area data which revealed how housing conditions and traffic safety varied across different areas and demonstrated how environmental factors were linked to chronic disease death rates.

### Interoperability of public health data systems

The management of multiple indicator datasets requires advanced technical solutions to handle their complex nature. Currently, there are several public health databases worldwide which aim to compare health conditions in various countries. Surján *et al.* [22] established a core ontological model that utilized semantic web technology to create a formal system which united 19 indicators between the WHO Health for All Database, OECD, and ECHI databases. Through the system, indicators could link to common dimensions which included time, space, gender, and disease. The model demonstrated potential for increased interoperability but had a limited capacity to process dynamic relationships and relational complexity.

### Health system performance

Health systems utilize indicators to assess their performance. Gaeta *et al.* [23] analysed Beveridge, Bismarck, and Mixed systems through ten performance indicators including mortality rates, vaccination coverage, and health expenditure. All models demonstrated better life expectancy along with reduced mortality rates, however, economic investments proved to be the primary performance-determining factor. Despite the fact that the research could not identify the best performing health system, the multivariate analysis indicated that the best performing countries were those in which the health expenditure was higher in absolute terms, regardless of their health system.

### Scientific equity and research visibility

Chinchilla-Rodríguez *et al.* [24] highlighted how public health research visibility and impact differ between Latin American countries. Although Brazil and Mexico produced the most research in the region, their global recognition was limited because they worked with fewer international partners while favoring publications in their native language. The countries of Peru, Uruguay, and Puerto Rico achieved better citation impact through their participation in international and English-language publication activities. The INIQUIS index was used to measure inequality, however, researchers discovered no regular relationship between research production and population wellness. Systemic challenges in language barriers along with dissemination problems and collaboration restrictions act as major obstacles to successful knowledge sharing.

## Discussion

This scoping review highlights the critical role of public health indicators in population health monitoring and policymaking. Drawing on multiple perspectives regarding indicator definitions, implementation methods, and assessment techniques across regions and systems, the review underscores that while frameworks like ECHI and EUHPID are well-designed and effective, their implementation remains uneven due to data limitations, funding gaps, and governance challenges. The thematic analysis reveals that existing indicators are not harmonized—particularly at subnational levels—and often fall short of ethical and methodological standards. Variability in presentation techniques, system interoperability, and regional adaptation further limits their effectiveness. Ultimately, although public health indicators are essential for comprehensive and real-time health system evaluation, they are not yet fully optimized, and their successful application depends on resolving tensions between standardized approaches and context-specific needs, as well as ensuring technically sound and meaningful interpretation.

By delivering descriptive data, health indicators enable strategic decision-making and support the monitoring of organizational accountability. The European Community Health Indicators (ECHI) represents one of the most sophisticated systems to achieve health information standardization among different countries [18]. The ECHI project established a multilevel indicators system, however, the EU requires a permanent health monitoring and reporting system. National authorities face challenges in adopting indicators because of limited data availability and institutional funding after EU economic support ends. Health systems encounter institutional and political challenges that prevent the implementation of indicators frameworks in their everyday practice.

Several studies emphasized ethical considerations of indicator development which requires balancing methodological soundness with values such as equity, transparency, and accountability. Etches *et al.* [10] demonstrated how indicators need to operate through frameworks that support public health’s fundamental values including equity, justice, and transparency. The entire process of indicator selection and dissemination automatically promotes specific knowledge systems while giving priority to particular policy directions. Indicator design needs to combine methodological soundness with ethical thoughtfulness for proper development. Similarly, the review of Freeman [27] highlighted that technical problems regarding indicator selection lie within availability, validity, and reliability of available data.

Flowers *et al.* [19] provided a detailed analysis of indicator presentation and interpretation issues which supports the findings of other research studies regarding health performance metrics [28, 29]. Use of inappropriate indicators can be misleading and can result in negative consequences for public health. The authors provide better alternatives through their proposal of statistical process control and funnel plots to enhance the assessment of random variation and uncertainty. The development of indicator literacy needs to be strengthened across different stakeholders who create and utilize these metrics.

The review highlights that health indicator usage has gradually shifted its emphasis towards subnational and urban levels in addition to national measurements. The ISARE II project together with the EURO-HEALTHY initiative demonstrated how public health stakeholders understand determinants of health at local and regional levels [21]. The implementation of regional indicators encountered difficulties with definitional inconsistencies, insufficient data, and classification methods. These initiatives demonstrate that health governance must incorporate national standards while allowing for regional adaptations.

Technical initiatives attempted to develop indicator systems which would integrate data from various sources. The ontology-based model developed by Surján *et al.* [22] demonstrated an innovative strategy to standardize indicator definitions between databases through semantic web technologies. Such systems offer strong conceptual value and technical potential, however, face implementation challenges. The adoption of semantic modeling technology demonstrates potential to achieve data standardization and system interoperability in the modern policy environments filled with data.

The evaluation of health system performance through indicators provides additional useful comparative features as demonstrated in the research by Gaeta *et al.* [23]. The research presents contradictory findings as economic investments instead of structural elements appear to provide better population health results. The research confirms that, as expenditures play a crucial role, so total health expenditure is important for a good performing health system.

The research conducted by Chinchilla-Rodríguez *et al.* [24] demonstrated that dissemination of scientific knowledge remains unequal as research visibility mainly depends on language and collaboration networks. The prevalence of English language publications creates obstacles for researchers working in other languages which restricts the inclusion of diverse contributions in widely used indicator frameworks. Public health indicators need to address inequalities which include research output imbalances between various geographical regions as these factors influence global policy decision-making and resource allocations. The findings of this review are consistent with those of Ramlackhan *et al.* [30], who emphasized the importance of non-peer-reviewed literature regarding the development of quality indicators and underscored the need for more transparent documentation of framework and indicator development processes.

The included studies present an extensive view of both strengths and weaknesses of indicators used in public health practice. These indicators serve as fundamental tools for modern health governance; however, they emphasize the need to evaluate their selection and interpretation methods. The effectiveness of indicators depends on their technical quality, their successful integration into different health systems and their ethical compliance. Indicators must adapt their capabilities to detect health threats such as climate change and pandemics which are transforming the worldwide public health environment.

This review contains multiple strengths. It utilizes literature from empirical research and policy reports that cover different countries and geographical areas. The analysis unites research findings about indicator methods with system performance assessments to present a detailed overview of current indicator development and application practices. However, the study contains several limitations. The included research consisted of English-language resources which could have omitted valuable studies from non-Anglophone regions. The review identifies implementation difficulties, however, fails to measure indicator adoption rates and their policy-level effects. Future research needs to investigate how indicators affect long-term public health results and create coherent indicator development methods that involve various healthcare systems globally.

Through a structured analysis of eleven peer-reviewed studies and organizational reports, this review highlighted key developments in public health quality indicators. The development and implementation of indicators for public health surveillance and policy evaluation remains complex despite their essential role as monitoring tools. The development of effective indicators requires finding a balance between scientific precision and effectiveness while considering both moral principles and environmental factors.

## Conclusion

Public health indicators serve as essential tools for health systems monitoring and policy development, however, their implementation so far shows inconsistent results. Current indicators have restricted applicability in practice as they demonstrate varying levels of methodological quality and policy significance.

The current public health indicator frameworks meet essential requirements, however, their capacity to address the multifaceted challenges of contemporary public health remains limited. Research demonstrates that public health indicators require continuous investment regarding their technical infrastructure and conceptual frameworks. The evolution of indicators requires both methodological advancement, continuous political support and worldwide partnership. Future research should include indicator policy impact assessment, framework improvement, and real-time public health assessment.

Conflict of interest: The authors declare no conflict of interest. The authors affiliated with the World Health Organization are solely responsible for the views expressed in this publication, which do not necessarily represent the decisions or policies of the WHO.

## Data Availability

The data underlying this article will be shared on reasonable request to the corresponding author. Key points Public health indicators are important tools for population monitoring and guiding evidence-based decision-making. Standardized public health indicator frameworks improve data collection process across various countries. Subnational monitoring initiatives highlight challenges in data availability, comparability, and implementation of public health indicators. The integration of ethical considerations during indicator design becomes essential to achieve fairness alongside transparency and policy relevance. The development of effective public health policy and system evaluation depends mainly on strengthening indicator infrastructure and governance systems at both national and regional levels.
